# ASO Author Reflections: From Susceptibility Loci to Oncologically Relevant SNPs in Upper Gastrointestinal Cancer

**DOI:** 10.1245/s10434-021-10819-z

**Published:** 2021-09-30

**Authors:** Jin-On Jung, Christiane J. Bruns, Thomas Schmidt

**Affiliations:** Department of General, Visceral and Transplantation Surgery, University of Cologne, Cologne, Germany

## Past

A vast number of genome-wide association studies in the current literature have reported many susceptibility loci for gastroesophageal cancer and its risk factors.^1^ As the number of allegedly relevant single nucleotide polymorphisms (SNPs) increases, it becomes harder to maintain an overview of those SNPs that might also have a clinical significance and lead back to molecular pathways of oncologic relevance. Therefore, an urgent need exists for a translational approach to build a bridge from genomic data on molecular expression to long-term oncologic prognosis. This type of approach could eventually lead to a better understanding of tumor biology and recurrence. This study aimed to find clinically relevant SNPs that may have a direct impact on oncologic outcome.

## Present

This study grouped 190 patients with curative oncologic resections of gastric and distal esophageal adenocarcinomas by SNPs previously linked to gastroesophageal cancer, Barrett esophagus, or gastroesophageal reflux disease (GERD) and analyzed for outcome differences.^2^ The study was able to set the focus on two SNPs (rs12268840 and rs9972882) that showed significant differences in overall survival and also were expression quantitative trait loci (eQTL) for the genes *MGMT* and *PGAP3*. Group TT of rs12268840 had the highest rate of second primary carcinoma, the lowest expression of *MGMT* in normal gastroesophageal tissue, and the worst oncologic outcome. Likewise, group AA of rs9972882 had the highest rate of distant metastases, the highest expression of *PGAP3* in healthy tissue, and the worst oncologic outcome.

## Future

The results of the study suggest that only a fraction of previously reported SNPs may also have a molecular or clinical impact on patients with upper gastrointestinal cancer. Although the results of this study should be verified in vitro*,* it is already clear that an integrated analysis of all available data is necessary to identify those genomic loci that are significantly related to oncogenesis and tumor progression.

The Cancer Genome Atlas has elucidated the genetic characteristics of upper gastrointestinal adenocarcinoma and has led to a comprehensive molecular subclassification.^3^ However, it currently is essential to make use of the detected genomic differences to trace them back to molecular and hopefully clinical implications. In this context, the analysis of eQTL has helped to provide a better understanding SNP data,^4^ although the correlation with oncologic outcome is only fragmentarily available in the existing literature.^5^

At a time when data become growingly complex, the identification of relevant genomic loci may sometimes resemble a search for the needle in a haystack. However, this could be simplified via the presented steps of narrowing down the available data. Another possible option for tackling this question could be the analysis of SNPs also found to be eQTLs for molecular agents involved in angiogenesis or other oncogenic pathways. With the advances in computational methods, it will furthermore be possible to analyze big genomic datasets directly. Thus, machine-learning methods could be used to analyze genomic data without any selection bias caused by expert opinion or a manual literature search. In the future, the demand for clinical professionals with simultaneous knowledge of advanced and integrated analyses will rise as the amount of data grows.
